# Longitudinal Association Between the Quality of the Separated Parents’ Relationship and the Frequency of Father–Child Contact: The Mothers’ Perspective

**DOI:** 10.1177/0192513X231226149

**Published:** 2024-01-04

**Authors:** Sylvie Drapeau, Karl Larouche, Hans Ivers, Sarah Dussault, Amandine Baude

**Affiliations:** 1 École de psychologie, 204255 Université Laval, Québec, QC, Canada; 2 Département de psychologie, 586079 Université de Bordeaux, Bordeaux, France

**Keywords:** separation, parents’ relationship, father–child contact, longitudinal study

## Abstract

This longitudinal study is based on family systems theory and aims to explore the association between the quality of the separated parents’ relationship and the frequency of father–child contact up to five years after parental separation. Using data collected from 408 families from the Quebec Longitudinal Study of Child Development (QLSCD), multilevel analyses and latent growth curve model were carried out. The results highlight a positive association between the separated parents’ relationship and father–child contact and demonstrate the impact of the initial contact frequency on the evolution over time of the separated parents’ relationship. They also highlight the contribution of custody tension, the child’s age, the length of time the couple lived together, and the socio-economic status on the initial levels of the studied trajectories.

## Introduction

In the Province of Quebec, Canada, where this study took place, 33% of children under the age of twelve had parents who separated (Desrosiers et al., 2018), with similar estimations found in the United States and European countries (Andersson et al., 2017). Even though the number of separations and divorces has been stable over the last decades, the structure of post-separation families continues to diversify (Castagner Giroux et al., 2016). One of the changes observed at the demographic level has been the growing involvement of fathers in the lives of their children (Westphal et al., 2014). A Canadian study found that, in the two years following the separation, 70% of the children maintained regular contact with their two parents (Juby et al., 2005). Furthermore, the proportion of Canadian, American, and European families in joint physical custody is increasing, even though sole maternal custody remains the most common arrangement (Bala et al., 2017; Meyer et al., 2022; Sodermans et al., 2013). A situation in which the two parents remain involved challenges them to put aside their former identities and roles as partners while maintaining their interdependent roles as parents (Jiminez-Garcia et al., 2019).

Parent–child and mother–father relationships are key factors in families’ and children’s well-being after the parental separation (Beckmeyer et al., 2021). According to family systems theory, these relationships are two interrelated subsystems, exerting a reciprocal influence on each other (Cox et al., 2011). Studies that have examined the interdependence between these two subsystems have some limitations. On the one hand, these studies have largely been conducted with samples of non-resident fathers with the Fragile Families in Child Wellbeing Study (FFCWS) dataset, thereby over-representing non-marital birth and socioeconomically disadvantaged families (Carlson et al., 2008; Fagan & Palkovitz, 2011, 2019; Goldberg, 2015). Although these studies have provided valuable insights, their focus is not per se on family reorganization after the couple’s break-up, even though separated parents, married or not, face unique challenges. This includes the need for each partner to adjust themselves to the dissolution of their couple relationship and to the redefinition of the roles it requires, that is, from a couple to parents only (Jiminez-Garcia et al., 2019). This can lead to various degrees of acrimony and negativity between the former couple, especially in the period surrounding the separation that is considered particularly important to the quality of their relationship as parents (Ferraro et al., 2018; Petren et al., 2017). On the other hand, these studies used variable-centred analyses (based on group means) to shed light on the direction of the association between the mother–father and father–child subsystems from two or three measurement points (Carlson et al., 2008; Fagan & Palkovitz, 2011, 2019; Goldberg, 2015; Petren et al., 2020). Although relevant, these studies do not examine how each of these subsystems evolves over time or how the trajectory of one co-varies with that of the other, which would be possible through person-centred analyses (e.g. growth curve analysis). To the best of our knowledge, only one study has examined this dynamic longitudinal association over time using the FFCWS dataset with this approach (Mallette et al., 2020). To address these gaps, the present study explores the interdependence of the trajectories of the quality of the separated parents’ relationship and father–child contact up to five years after the couples’ break-up.

### Conceptual Framework

Family systems theory is widely used by family scholars to explain how parental separation affects family relationships (Demo & Buehler, 2013). A couple’s break-up can destabilize the family system to varying degrees, and the first years are considered a key period in the redefining of family relationships (Ahrons & Miller, 1993; Hetherington, 2003). After the separation, even though the parents and children live in two households, the parents still maintain a relationship based on their child, as in binuclear families (Ahrons, 1979). One of the most difficult tasks facing separated parents is that of redefining their relationship in order to fulfil, as coparents, their shared childrearing responsibilities. The former partners’ difficulty in moving away from their roles and identities as spouses to those of coparents is likely to be greater when there is a great deal of emotional intensity (e.g. sadness, anger) towards one another or when there is difficulty in accepting the separation (Sbarra & Emery, 2008). Above and beyond the parent–parent relationship, the couple break-up also has profound implications for the parent–child relationship, especially for the fathers who generally do not take on the primary caretaking role. In a non-resident context, fathers encounter many challenges to remaining involved in their children’s lives (Fagan & Kaufman, 2014).

A key tenet of family systems theory is that family subsystems are interdependent, exerting a continuous and reciprocal influence on each other (Cox & Paley, 1997). What takes place in the parent–child dyad is influenced by the parent–parent subsystem, and vise-versa (Arditti & Kelly, 1994). According to this interdependence principle, the separated father’s commitment to a child has the potential to influence the quality of the coparenting relationship. This principle also implies that the ex-partners’ relationship influences the relationship between the non-resident father and the child (Braver et al., 2005).

### Literature Review

Past research has shown various trajectories that can represent both the separated parents’ coparenting relationship and the frequency of father–child contacts. Regarding the separated parents’ relationship and based on group means, authors in the field generally propose a linear portrait of the situation: there is a high degree of tension between separated parents at the beginning of the transition, the intensity dropping off progressively over time (Emery, 1999; Maccoby et al., 1992). Using growth curve analysis to capture change over time, recent studies have depicted a similar trajectory based on different samples and different indicators for the separated parents’ relationships. For example, by interviewing mothers five times in the year following the divorce (*N* = 135), Hardesty et al. (2017) illustrate an average downward trajectory of inter-parental conflict, communication, and harassment, but no change in coparental support. This study only covers the first year after the break-up and does not make it possible to observe improvement in the parental relationship after the initial period of instability. Using the FFCWS, two other studies of unmarried parents who separated when the child was young show a linear decline in parental cooperation over the first 5 years of the child’s life (*N* = 1603) (Dush et al., 2011) or as the child went from 3 to 9 years (*N* = 1193) (Goldberg & Carlson, 2015).

Regarding the frequency of father–child contact, the first wave of research also proposed a linear portrait of the changes in the fathers’ contact with their children which declined gradually over time (Furstenberg & Cherlin, 1991). To our knowledge, the American study of Cheadle et al. (2010) was the first to have documented fathers’ trajectory patterns using growth mixture modelling. These authors reported that, on average, the frequency of father–child contact decreases gradually over time to stabilize after about 10 years. That said, the majority of the families were characterized by a stable trajectory that was marked by infrequent contact (32%) all throughout the investigated period or, on the contrary, frequent contact (38%).

Thus far, most studies addressing the directionality of the association between mother–father and father–child subsystems in families where the father does not live with the child are based on cross-lagged or path-analysis models. Taken together, these studies suggest that the father–mother and the father–child subsystem are interdependent; they show that each of these subsystems can be a precursor to the other. For example, based on a sample of 184 divorced parents recruited from divorce requests (children were on average 8 years old), the results of Petren et al.’s study (2020) show that the frequency of the father’s involvement in various activities at time 1 (about 3 months post-divorce) predicted cooperation in coparenting at time 2 (from 3 to 6 months later) but not the reverse. The results of studies based on the FFCWS also point to a bidirectional relation between coparental cooperation and the non-resident fathers’ involvement, (Carlson et al., 2008; Fagan & Palkovitz, 2011, 2019; Goldberg, 2015). Nonetheless, coparenting more strongly predicts the non-resident fathers’ involvement than does the opposite. To the best of our knowledge, only one study has examined the dynamic longitudinal association between these subsystems over time (Mallette et al., 2020). Using a sample of 1623 mothers from the FFCWS, Mallette et al. (2020) noted that the coparenting relationship and father involvement have concurrent influence on each other over the child’s first 5 years. Their sample included unmarried mothers, about half of whom had not had a romantic relationship with the father or cohabited together. Characteristics of this sample limit generalization to parents in post-separation, notably those who were legally married before the transition.

### Characteristics Associated with the Father–Mother and Father–Child Subsystems

Numerous parent-related, child-related, and separation-related characteristics have been associated with the father–mother and father–child subsystems. In terms of parental characteristics, the lower socio-economic status and older age of the mother is associated with a lower frequency of contact between the non-resident father and his child (Cheadle et al., 2010). In terms of child characteristics, parents of boys have a poorer coparenting relationship (Goldberg & Carlson, 2015), but fathers are more involved with their child (e.g. time, activities, closeness) (Mitchell et al., 2009). Research also shows that non-resident separated fathers have fewer contacts with their child if the latter is older (Amato et al., 2009). Regarding separation-related characteristics, parents who were married before separation are more supportive of each other in their coparenting relationship over time (Dush et al., 2011) and divorced fathers have more contacts with their child than fathers whose child is born out of wedlock (Amato et al., 2009; Cheadle et al., 2010). The length of the parents’ previous relationship would also seem to be linked to the coparenting relationship, with a longer relationship being associated with more support between the parents and more contact between the father and children (Amato, 2009; Cheadle et al., 2010). Finally, tension in divorce proceedings and dissatisfaction with the financial arrangements for the child have been associated with a poorer coparenting relationship (Bonach, 2005).

### The Current Study

Founded on family systems theory, the aim of the present study is to explore the longitudinal association between two trajectories, namely, the quality of the separated parents' relationship and the frequency of father–child contact. Using a person-centred analytical approach, it sheds light on the reorganization of the family system up to 5 years after the couple’s break-up. This period captures the first years after transition, identified as crucial in terms of reorganizing family ties, and then the period when the family system may reach a new equilibrium. Based on a population sample, the study has the following specific objectives, namely: (1) describe the trajectories for the quality of the separated parents’ relationship and the frequency of father–child contacts up till 5 years after the couple’s break-up, (2) identify the separation-related (i.e. length and type of the previous relationship, custody tension), parent-related (i.e. age, socio-economic status), and child-related (i.e. age, sex) characteristics associated with these trajectories, and (3) explore their interdependence over time by examining a) the association between the intercepts and the slopes of the quality of the relationship and of the frequency of father–child contact; b) the predictive role of the initial levels (intercept) of the quality of the separated parents' relationship and of the father–child contact on the slope of the other variable. Since this is, to the best of our knowledge, the first study to examine the longitudinal interdependence between these two subsystems after a couple break-up, no hypothesis can be made at this time. The data comes from the Quebec Longitudinal Study of Child Development (QLSCD), a population-based representative sample of children in the Province of Quebec, Canada (born between October 1997 and July 1998). The sample is diversified in terms of father–child contact, including cases where contact was rare or absent and indicated paternal withdrawal, to those where the child divided his or her time equally between the two households (50% of the time). These situations reflect a reality that is increasingly widespread in the Western World (Steinbach et al., 2021), particularly in Quebec and Canada (Pelletier, 2016). Similarly, for the sake of diversity, the sample included situations where the separated parents were legally married or in common-law relationships, which is a common and sizeable social reality in the Province of Quebec (Desrosiers et al., 2018) and which is now increasing worldwide (Chamie, 2017). The potential for the generalization of the present study is therefore high.

## Method

### Population

The ongoing QLSCD study is based on a representative sample of children born to mothers residing in Quebec in 1997–98 with the exception of extremely premature babies and the health regions of Northern Quebec, Cree Territory, Inuit Territory, and Native reserves (*N* = 2120). Between 1997–98 and 2015, follow-ups were conducted with the children on an annual basis from five-months up till the age of eight and then every two years up to 17, for a total of 14 rounds (each survey year was called a ‘round’). The high frequency of the data collection of the QLSCD is one of its distinguishing features compared to other longitudinal surveys of families elsewhere in the world (Pelletier, 2016). The QLSCD makes it possible to study family reorganization longitudinally and to target the period proximal to the break-up as the starting point for the trajectories. That said, information about the children and their family context came from several sources (e.g. target child, parents, teacher), but for the most part came from the ‘person most knowledgeable about the child (PMK)’, which is the mother in 99% of cases in the QLSCD (Jetté & Des Groseilliers, 2000) and all cases in this study.

### Sample

The study focused on a sub-sample of the QLSCD, namely, children whose parents had separated (*n* = 637). In each round, whether or not a separation occurred was determined by the answer *Yes* or *No* to the following question: ‘Since the interview on [date], did [child’s name]’s parents break-up and stop living together?’ If the answer was *Yes*, the respondent indicated the date the separation took place. The exclusion criteria were applied in the following order: 1) one parent had died (*n* = 8); 2) the father had sole custody for at least one round (*n* = 94); 3) the couple’s physical separation occurred when the child was under 2 years old (round 3) or over 16 years old (round 13) (*n* = 118). A fourth exclusion criterion was applied when there was a complete absence of data for both dependent variables (*n* = 9). Situations where fathers have sole custody were excluded in order to remain in line with the research objective. The frequency of father–child contact is no longer an issue when the child lives with the father. Moreover, families whose child was under 2 years old were excluded because the challenges experienced by parents of very young children at the time of separation are specific to this age group (McIntosh et al., 2010). Regarding families whose child was over 16 years old, they were excluded to limit families that could only have complete one round without their child being 18 years old. Once the exclusion criteria were applied, 408 families were selected for analysis. The present study focuses on the five years following the separation, which occurred between 2000 and 2013. As the parents separated at different points across these rounds, the number of observations available for each participant varied.

Prior to the separation, the parents were together on average 11.4 years (*SD* = 5.5; ranged from 2 to 34 years); of these, 58.4% (*n* = 238) were in a commow-law union, which is representative of Quebec families (*Institut de la statistique du Québec*, 2020). At the first post-separation measure, parents had been separated for an average of 9.2 months (*SD* = 12.7), 84.3% (*n* = 344) of them had been separated for 12 months or less (*Mdn =* 5.0 months) and about 37% of mothers (*n* = 98) reported being in a new relationship. The children’s age when the separation was reported varied from 2 to 16 years old (*M* = 7.4, *SD* = 4.1); of these, 49.5% (*n* = 202) were girls. At the time of separation, the mothers’ age varied from 20 to 55 years old (*M* = 34.6, *SD* = 6.8). Fathers’ age varied from 21 to 58 years old (*M* = 37.4, *SD* = 6.8). As for their household annual income, 14.0% (*n* = 57) earned from 0 to $19,999, 29.0% (*n* = 118) from $20,000 to $39,999, 29.6% (*n* = 121) from $40,000 to $59,999, and 24.3% (*n* = 99) more than $60,000. A university degree was held by 26.5% (*n* = 108) of mothers, a community or vocational college diploma by 34.8% (*n* = 142), a high school diploma by 23.8% (*n* = 97), and no diploma at all by 12.3% (*n* = 50).

### Measures

#### Quality of the Separated Parents’ Relationship

At the time of separation and at subsequent collection rounds, mothers answered the following question to describe the quality of their relationship with the child’s father: ‘How would you describe the CURRENT situation between you and the biological father of your child?’: 1 = *good*, 2 = *fairly good*, 3 = *bad*, 4 = *very bad*. This variable was recoded as ‘4=good to 1=very bad’ (when an answer was not given, another question was asked only for the round where the separation occurred: ‘If you have separated from the biological father of your child SINCE OUR LAST VISIT A YEAR AGO, how would you describe the emotional atmosphere surrounding this separation?’). At the first measure, 29.4% of respondents described the relationship as ‘good’, 42.8% as ‘fairly good’, 17.6% as ‘bad’, and 10.2% as ‘very bad’.

#### Frequency of Father–Child Contact

At the time of separation and at subsequent collection rounds, the respondents reported who the child lived with and the contact the child had with the other parent according to the following scale: 1 = No physical contact with the father, 2 = Sporadic contact, 3 = [child] sees his father every two weeks, 4 = [child] sees his father every week, 5 = Shared physical custody, with more time living with the mother, 6 = Equal shared physical custody. At the first measurement time, 32.9% of the children were living in equal shared physical custody, 14.2% were living in shared physical custody, with more time living with their mother, and 52.9% seeing their father according to different arrangements: every week (18.0%), every two weeks (17.7%), sporadic contact (11.9%), and no physical contact with their father (5.3%).

#### Characteristics Related to Context

The tension created by living arrangements and visiting rights is measured by the following question: ‘Between the [target-child’s] parents, is the question of living arrangements and visiting rights 1 = *no source of tension at all*? 2 = *very little source of tension?* 3 = *some source of tension?* 4 = *a big source of tension?*’. At the first measurement time, 53.7% of respondents reported no tension, 21.3% very little tension, 12.5% some tension and 9.6% reported a lot of tension. The following variables were also used: the pre-separation relationship status (married or common-law relationship) and the length of the parents’ pre-separation relationship.

#### Characteristics Related to Parents

A composite indicator, created by the QLSCD based on parental education and household income, was used to assess the socio-economic level according to a normalized score (Z). The mother’s age at the time of separation was documented; the father’s age, not being measured at the time of separation, was estimated by adding the child’s age at the time of separation to the father’s age at the time of the child’s birth.

#### Characteristics Related to the Child

The respondents reported the child’s age in months at the time of separation as well as the child’s sex. For the moderation analyses, three age groups were formed: under 5 years (*n* = 179); 6–10 years (*n* = 128); 11–16 (*n* = 101) years.

### Data Analysis

First, in order to model the trajectories for the quality of the separated parents’ relationship and the frequency of the father–child contact (objective 1), longitudinal, ordinal (cumulative logit), multilevel analyses were conducted. Based on this type of analyses, all of the family-specific observations can be conceptualized as a trajectory, which has a single starting point (the intercept, i.e., the value of the dependent variable at the time of separation) and a slope (i.e. the amount of change in the dependent variable per unit of time, that is, any of the given years in this study) (Singer & Willett, 2003). A multilevel model allows these two parameters (starting point and slope) to be random variables whose values can vary from one family to another (Brown & Prescott, 2014). In the context where these analyses were performed on ordinal variables (logistic model), these two coefficients are used to estimate the risk of moving to the higher response category of the dependent variable at the time of separation or for each passing year.

Second, in order to document the association between the two trajectories and contextual, parental, and child characteristics (objective 2), some variables were added to the models used in objective 1. The introduction of these variables into the model, as a main effect and in interaction with time, made it possible to estimate their moderating role on the intercept and the slope.

Finally, a structural equation approach modelling the relationships between the trajectories for the quality of the separated parents’ relationship and the frequency of father–child contact (bivariate latent growth curves) was estimated to document their possible longitudinal interdependence (objective 3a and b). These analyses provide estimates of the covariance between the intercepts and slopes of the dependent variables.

The analyses of objectives 1 and 2 were performed via SAS software (version 9.4, SAS Institute, 2014), while analyses of objective 3 were performed via the Mplus version 7 software package (Muthen & Muthen, 2012), using a two-tailed test with a 5% level of significance. All analyses were conducted using normalized longitudinal weights estimated in the QLSCD, so as to maintain the representative nature of families in Quebec.

## Results

### Missing Data

Taking into account that the number of observations available for each respondent varied as parents separated at different points across the rounds, and that young people were no longer followed after the age of 18, 1844 observations were potentially available in the database. Due to the non-participation of some respondents, in at least one survey round during the 5-year follow-up of the present study, 1565 of these 1844 possible observations were accessible in the QLSCD database (84.9%).

Among the 408 families, 72% of respondents had at least 3 points of observation, 20% had 2 and 8% of respondents had 1. Respondents who had only 1 point of observation were not excluded because their data still contribute to (1) the estimated mean at the first time point, (2) the estimated contribution of predictors at the first time point, (3) the estimated residual variability of the dependent variables (which is used to estimate the standard error of all model parameters and is the divisor for most statistical tests), and (4) increase the number of degrees of freedom and therefore the statistical power.

Of the 1565 accessible observations, 1383 (88.4%) had answered for both dependent variables. Exploratory analysis revealed that the probability of not having completed the dependent variables was associated with having a younger child (*p* < .05), having a lower socio-economic status (*p* < .05), being in a relationship for a shorter period of time prior to separation (*p* < .05), and the father being younger (*p* < .05).

### Trajectory and Moderators of the Quality of the Separated Parents’ Relationship

First, an exploratory model with only one random effect (intercept) revealed the presence of significant variability in the families’ mean relationship quality, *X*^*2*^ (1) = 265.17, *p* < .01, thus justifying the need to use a multilevel model. Second, a longitudinal multilevel model including random intercept and quadratic time effects (including the linear effect) was estimated to establish the shape of the trajectories. The results reveal the absence of a significant linear effect for the time since the separation on the quality of the parents’ relationship, F(1,289) = .43, *p* = .51, but support the presence of a quadratic relationship, F(1,207) = 3.60, *p* = .05, indicating that the parents’ relationship tended to be of lower quality in the first two years after separation and then improved significantly. Descriptive statistics revealed that, during the first year post-separation, about a third of respondents (35.9%) report a good relationship with the child’s father. Two years after the separation, this number increased to 42.8% and reached more than half of respondents (51.8%) five years after the initial separation. However, only the intercept and the linear time effect seemed to vary between the families, as the variance of their random effects being significant at *p* < .001. Based on these results, the final model to study the predictors was set up as a longitudinal multilevel model with the intercept and quadratic slope, which includes by default the linear slope, with the intercept and linear slope being random effects.

Among the eight characteristics studied in the final model, a significant moderating effect of the intercept (i.e. relationship quality at the time of separation) was observed for three of them: the children’s age group, F(2,346) = 4.22, *p* = .02; the length of time the couple had lived together prior to separation, F(1,346) = 4.96, *p* = .03; and custody tension, F(1,346) = 54.17, *p* < .001. Specifically, compared to the parents of teenagers (11–16 years), the parents of children aged 6–10 years and under 5 years were, respectively, 2.74 and 6.05 times more likely to report a better quality of relationship with the other parent at the time of separation. Furthermore, each additional year of living together was associated with a 11% (OR = 1.11) increase in the odds of reporting better relationship quality at separation. Finally, each one-level *decrease* in tension (on a scale of 1–4) was associated with a 3.44-fold increase in the odds (OR = 3.44) of reporting a better relationship at separation. No significant moderating effects of the intercept were found for the child’s sex (*p* = .57), type of couple relationship (*p* = .48), socio-economic status (*p* = .51), age of the mother (*p* = .31), and age of the father (*p* = .73). For their part, the trajectory slopes did not seem to be moderated by the eight characteristics studied here. Indeed, the results indicate no significant moderating effect of these characteristics on the trajectory for the quality of the parents’ relationship in the five years following separation: the child’s sex (*p* =. 24), child’s age (*p* = .62), socio-economic status (*p* = .68), length of time the couple lived together prior to separation (*p* = .97), mother’s age (*p* = .55), father’s age (*p* = .81), custody tension (*p* = .20), and couple relationship type (*p* = .57).

### Trajectory and Moderators of the Frequency of Father–Child Contact

First, an exploratory model with only one random effect (intercept) revealed the significant variability in families in the mean frequency of father–child contact, *X*^*2*^ (1) = 54.33, *p* < .01, thus justifying the need to use a multilevel model. Second, a longitudinal multilevel model with two random time effects (linear and quadratic) was estimated to establish the shape of the trajectories. The results show a significant linear effect of time since the separation on the frequency of father–child contact, F(1,268) = 17.73, *p* < .001, as well as a more modest quadratic relationship, F(1,107) = 7.31, *p* = .08, suggesting that the frequency of father–child contact decreased in the first three years and then stabilized. During the first year after the separation, about a quarter of respondents (27.7%) report that the child has contact with their father once per two weeks (or less frequently) but this percentage increased to 56.0% after three years. Five years after separation, 55.0% of respondents were still reporting father–child contacts less than once per two weeks. However, only the intercept and linear effect of time seemed to vary among families, their random effects being significant, *p* < .001. Based on these results, the final model to study the predictors was set up as a longitudinal multilevel model with the intercept and quadratic slope, which by default included the linear slope, with the intercept and linear slope being random effects.

Among the eight characteristics studied in the final model, only two showed a significant moderating effect of the intercept (i.e. the frequency of father-child contact at the time of separation): socio-economic level, F(1,106) = 6.55, *p* = .01, and custody tension, F(1,106) = 14.34, *p* < .001. The mother’s age was marginally significant with *p* = .08. Specifically, the estimated odds ratio for the socio-economic level (OR = 1.98) suggests that each increase of one standard deviation in the socio-economic level was associated with a 98% increase in the odds of more frequent contact at separation. In addition, each decrease of one degree in tension (on a scale of 1–4) was associated with a 2.39-fold increase in the risk/odds (OR = 2.39) of reporting more frequent father–child contact at separation. No significant moderating effects of the intercept were found for the child’s sex (*p* = .13) or age group (*p* = .39), type of couple relationship (*p* = .81), length of time the couple had lived together before separation (*p* = .63), and the father’s age (*p* = .63).

For its part, the slope of the trajectory for the frequency of father–child contact did not seem to be moderated by the eight characteristics studied. Indeed, the results indicate no significant moderating effect for the following characteristics on the contact trajectory in the five years following separation: child’s sex (*p* = .18), child’s age (*p* = .21), socio-economic status (*p* = .96), length of time the couple had lived together prior to separation (*p* = .89), mother’s age (*p* = .43), father’s age (*p* = .66), custody tension (*p* = .66), and type of couple relationship (*p* = .51).

### Cross-sectional and longitudinal association between the trajectories for the quality of the separated parents’ relationship and frequency of father–child contact

**Figure 1.**
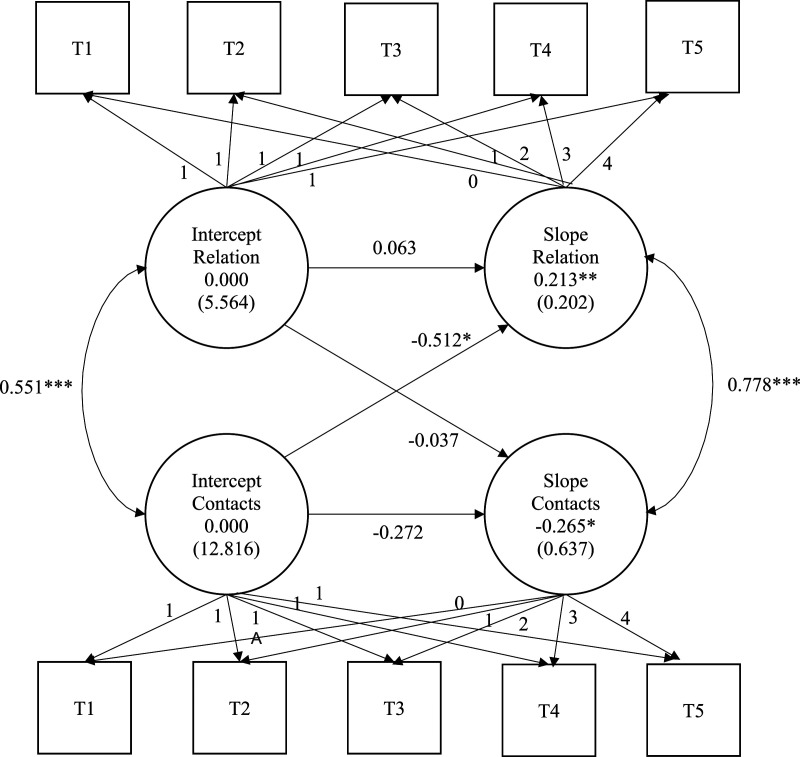
Ordinal bivariate growth model between parental relationship quality and frequency of father–child contacts. *Note*. Mean (variance) of latent intercepts and slopes are displayed. Mean intercepts are fixed at 0 since there are many intercepts (one for each modality, except the lowest reference modality) in ordinal models. *p < .05, **p < .01, ***p < .001.

A bivariate growth curve model was used to estimate the covariation between the random effects (intercept and slope) of the two trajectories (see Figure 1). As the ordinal (cumulative logit) model was estimated using numerical integration, no fit indices are available. After covariances between random effects were standardized, the results indicate (a) the presence of a positive and significant association between the quality of the parents’ relationship and the frequency of father–child contact at the time of separation, *B* = .551, *p* < .001, as well as a strong positive and significant association between the slopes of the two trajectories, *B* = .778, *p* < .001; and (b) a negative association between the intercept of the contact trajectory and the slope of the relationship quality trajectory, *B* = −.512, *p* = .03. However, the inverse relationship between the intercept of the relationship quality trajectory and the slope of the contact trajectory was non-significant, *B* = −.037, *p* = .82. No relationship between intercept and slopes within each trajectory was found significant, for relationship quality (*B* = .063, *p* = .76) and father–child frequency of contacts (*B* = −.272, *p* = .31). Put another way, the quality of the separated parents’ relationship and the frequency of father–child contact co-evolved positively over the five years following the couple’s break-up. Moreover, the initial frequency of father–child contact was associated with positive changes in the quality of the separated parents’ relationship over the following 5 years, but the opposite was not true.

## Discussion

Based on a longitudinal design, and the mothers’ reports, this exploratory study highlights three main findings. First, as other authors have shown (Ahrons & Miller, 1993; Hetherington, 2003), the first few years after the couple break-up are crucial and are characterized by a concomitant change in the parents’ relationship and in father–child contact. These results underline the importance of studying this reorganization dynamically and targeting as the starting point the period proximal to the break-up. Second, consistent with the family systems theory (Cox et al., 2011), the present study sheds light on the interdependence of family ties and, more specifically, on the role of the initial frequency of father–child contact on the evolution of the separated parents’ relationship over time. This result highlights the importance of physical custody-sharing decisions in the period immediately following the break-up (Nielsen, 2017). Third, certain characteristics were correlated with initial levels of father–child contact and the quality of the separated parents’ relationship, namely: custody tension, the child’s age, length of time together, and socio-economic status, which makes it possible to focus on the groups most at risk. However, only the custody tension was associated with both dependent variables. This is a risk factor on which it is possible to intervene in order to influence post-separation family paths and, ultimately, the child’s adaptation to this context.

More specifically, the results show that the quality of the separated parents’ relationship and the frequency of father–child contact co-evolved positively over the five years following the couple’s break-up, which is consistent with the result obtained by Mallette et al. (2020). The present study makes an original contribution to this field because it specifically targeted couple break-ups. In this study, the initial frequency of father–child contact was associated with positive changes in the quality of the separated parents’ relationship over the following 5 years, but the opposite is not supported by the results. This finding that the father’s involvement can predict the quality of the coparenting relationship was found by Petren et al. (2020) in a sample of recently divorced mothers using a cross-lagged design. The father’s consistently positive involvement with his children seemed to strengthen the mother’s confidence in his parenting skills (Fagan & Kaufman, 2014; Petren et al., 2017). This result must, however, be seen in the light of our sample’s characteristics where the majority of fathers see their child very frequently and where the relationship climate was evaluated fairly positively by the mothers. This is consistent with what has been previously reported regarding separated fathers, that is, those with a lower quality relationship with their child (i.e. contacts, closeness, and support) are less likely to be represented in surveys (Kalmijn, 2021).

The results of the present study are, however, partially consistent with those of Mallette et al. (2020) in terms of the bidirectionality of the association between the subsystems. Indeed, these authors observed that the initial level of coparental cooperation was linked to the evolution of the fathers' involvement, and vice versa. However, our results show a unidirectional relationship between the initial level of contact and the parents’ relationship trajectory. Above and beyond the families’ other differences, it is possible that the age range of the children focused on in the respective samples helps to explain this difference. According to family systems theory, research into family subsystems must pay attention to the children’s developmental stage (Dyer et al., 2018). In the present study, the relationship trajectories are examined when the target child was from 2.5 to 16 years old at the time of the break-up, while Mallette et al. (2020) studied these trajectories when the target child was from 1 to 5 years old. Contrary to older children and adolescents, contact between very young children and the non-resident parent could be more dependent on the quality of the parents’ relationship (Beckmeyer et al., 2021). This hypothesis is worthy of further exploration.

The results concerning the trajectories taken one at time also make a relevant contribution. They show that the quality of the parents’ relationship declines in the first two years after the break-up but improves thereafter. Once the anger and sadness have subsided, parents were able to focus on their children and compartmentalize their relationship as ex-spouses (Beckmeyer et al., 2021; Markham & Coleman, 2012). By focussing on analyses that account for intra-individual changes over time, this study is, to our knowledge, the only one to have quantitatively demonstrated a downward and then upward trajectory of the relationship over a 5-year period. The fluid, variable aspect of the relationship between separated parents would seem to be better documented by a few qualitative studies (Jamison et al., 2014; Markham & Coleman, 2012). More specifically, our results are similar to the ‘Bad to Better’ trajectory identified by Markham and Coleman (2012) in interviewing separated mothers sharing physical custody with the father. Despite a difficult period of adjustment at the beginning, the mothers in this trajectory reported an improvement in the relationship with their ex-partner.

In the present study furthermore, the child’s age at the time of the break-up was associated with the quality of the separated parents’ relationship, that is, the parents of younger children reported having a better relationship. During certain stages of their children’s development, such as the transition into adolescence, parents may experience more stress and be less cooperative with each other (Baril et al., 2007). As regards the relationship between the length of time the couple lived together and the quality of the relationship between the parents, the results are consistent with those of other studies that suggest that the characteristics of the parents’ relationship before the separation are associated with the relationship quality that will develop afterwards (Cooper et al., 2015).

As concerns the trajectory for father–child contact, our results are in keeping with the mean trajectory observed by Cheadle et al. (2010), namely, a decrease in contact during the first few years after the break-up, followed by a stabilization. The results of Cheadle et al. (2010) show that the mean decrease is mainly observed among the sub-groups of fathers who are the most involved with their children, that is, those who see them at least once a week. The vast majority of the sample in this study is composed of such situations. There are a number of barriers to frequent father–child contact, including distance between households, transportation costs, distance to school and childcare (Hawkins et al., 2006; Hetherington & Stanley-Hagan, 2002), which may explain why such contact decreases in the early years. That said, the overall picture observed in the sample is one of the separated fathers’ involvement in their children’s life. In this respect, it is in line with recent data, since an increase in the frequency of contact between non-resident fathers and their children has been observed in recent decades (Kelly, 2007). Our results also show that the frequency of father–child contact is positively correlated with the socio-economic status of the household, which is consistent with other research (Cheadle et al., 2010).

Tension surrounding custody and visiting rights was the only variable associated with both the quality of the parents’ relationship and father–child contact. This is a type of conflict unique to non-cohabiting parents which can be amplified in the context of couple break-up (Sbarra & Emery, 2008). For example, when one parent considers that the other parent was not involved with the children prior to the break-up, he or she may perceive the other’s demands for shared physical custody negatively. Acrimony between parents can also lead to tensions around custody, as the difficult emotions experienced in the context of a romantic break-up can have an ensuing effect on the perception of the other person as a parent (Sbarra & Emery, 2008). At the other end of the spectrum, a low level of tension surrounding custody and visiting rights may reflect harmonious post-separation family dynamics where the father’s involvement is consistent with beliefs about the essential role of both parents in the development and well-being of children.

### Limitations

Certain methodological limitations warrant consideration. First, considering the availability of measurements in the dataset we used (QLSCD), the father involvement measure was limited to father-child contacts. This indicator does not reflect all the components of paternal involvement. However, referring to Lamb’s typology (Lamb et al., 1987), face-to-face contact refers to the accessibility component (time the father is available), and indirectly, to the involvement component (involvement in activities with their children), since fathers may want to maximize time shared with their children (Fagan & Palkovitz, 2019; Hawkins et al., 2006). In addition, from the mother’s perspective, the frequency of contact can be more easily observable than the father’s involvement in child-related activities considering that she is not present (Mikelson, 2008). This also allowed us to include fathers in the sample who had little or no contact with their child. Second, the completion rate of the QLSCD for separated non-resident fathers was considerably lower than for fathers living with the mother and focal child; relying exclusively on mothers’ reports was therefore the best option. The fact that the study data comes entirely from the mother could cause a problem of common method variance and come with a perceptual bias as well, since the quality of the relationship with the other parent may tint the mother’s perception of the father’s involvement. Third, the variables associated with the trajectories were considered only at the time of separation. It is possible that time-varying predictors that were not considered may have been associated with the studied trajectories. Finally, even though this study is based on data from the general Quebec population, large longitudinal surveys founded on representative samples like the QLSCD must accept the compromise that comes with broadly focused, large-scale studies, namely, measures comprised of few items (such as those used to evaluate the two main constructs of the current study) so to reduce the demand on the participants and the risk of attrition.

## Conclusion and Implications

The goal of this longitudinal study, based on representative data for the population of the Province of Quebec, was to identify the trajectories for the quality of the parents’ relationship and the frequency of father–child contact, to determine the characteristics associated with them, and to document the interdependence of these trajectories. The results made it possible to identify and pay more attention to the families most at risk at the time of the break-up in terms of the parents’ relationship and father–child contact (older child, higher separation tension, shorter length of time spent living together, and lower socio-economic status). They also highlight the interdependence between the mother–father and father–child subsystems at the time of separation and in the following years. Each of these subsystems should thus be the focus of policies and interventions to ultimately support families’ adjustment to separation. Moreover, it seems that the time the father spends with his child at the time of separation is crucial, considering its link with the change in the quality of the parents’ relationship over the following years. Future research on the interdependence between the mother–father and father–child subsystems in the context of couple separation should include more comprehensive measures of these constructs (e.g. measure of coparenting) as well as the father’s perspective. Mixed quantitative-qualitative designs would also be useful in further investigating the meaning of relationship trajectories in the participants’ eyes.
